# Development, Characterization, and Bioactivity of Non-Dairy Kefir-Like Fermented Beverage Based on Flaxseed Oil Cake

**DOI:** 10.3390/foods8110544

**Published:** 2019-11-03

**Authors:** Łukasz Łopusiewicz, Emilia Drozłowska, Paulina Siedlecka, Monika Mężyńska, Artur Bartkowiak, Monika Sienkiewicz, Hanna Zielińska-Bliźniewska, Paweł Kwiatkowski

**Affiliations:** 1 Center of Bioimmobilisation and Innovative Packaging Materials, Faculty of Food Sciences and Fisheries, West Pomeranian University of Technology Szczecin, Janickiego 35, 71-270 Szczecin, Poland; emilia_drozlowska@zut.edu.pl (E.D.); paulina.siedlecka@yahoo.com (P.S.); mmezynska@zut.edu.pl (M.M.); artur-bartkowiak@zut.edu.pl (A.B.); 2 Department of Allergology and Respiratory Rehabilitation, Medical University of Lodz, Zeligowskiego 7/9, 90-752 Lodz, Poland; monika.sienkiewicz@umed.lodz.pl (M.S.); hanna.zielinska-blizniewska@umed.lodz.pl (H.Z.-B.); 3 Department of Diagnostic Immunology, Chair of Microbiology, Immunology and Laboratory Medicine, Pomeranian Medical University in Szczecin, 72 Powstancow Wielkopolskich Avenue, 70-111 Szczecin, Poland; pawel.kwiatkowski@pum.edu.pl

**Keywords:** flaxseed, oil cake, beverage, fermentation, non-dairy, kefir grains, kefir-like

## Abstract

Flaxseed oil cake (FOC) was evaluated as a potential substrate for the production of a novel kefir-like fermented beverage. Three variants containing 5%, 10%, and 15% (*w/w*) of FOC were inoculated with kefir grains and incubated at 25 °C for 24 h. After processing, beverages were stored in refrigerated conditions (6 °C) for 21 days. Changes in microbial population, pH, acidity, levels of proteins, polyphenolics, flavonoids, ascorbic acid, and reducing sugars were estimated. Additionally, viscosity, firmness, color, and antioxidant properties were determined. Results showed that lactic acid bacteria as well as yeast were capable of growing well in the FOC without any supplementation. During refrigerated storage, the viability of the microorganisms were over the recommended minimum level for kefir products. As a result of fermentation, the beverages showed excellent antioxidant activity. Because of the functional characteristics conferred to the FOC beverages, the use of kefir grains showed adequate potential for the industrial application. Therefore, this beverages could be used as a new, non-dairy vehicle for beneficial microflora consumption, especially by vegans and lactose-intolerant consumers.

## 1. Introduction

In the past years, there has been a growing interest to develop new functional food products. The consumption of fermented beverages is a worldwide trend because of their health-promoting effects and associated properties [1,2,3,4,5,6,7]. Fermented beverages could contain probiotic microorganisms, which are living microorganisms that when administered in adequate amounts in edible matrices confer health benefits on the consumers [2,8,9,10,11]. Many of these microorganisms have been identified as lactic acid bacteria (LAB) and are usually consumed in the form of fermented milks, yogurt, or kefir [7,9]. In general, most fermented products have been found to contain at least 10^6^ colony forming units (CFU)/mL, with concentrations varying depending on several variables such as the product’s region, age and time at which the products are analyzed or consumed [3,6,8,10]. The Food and Agriculture Organization (FAO) and the World Health Organization (WHO) suggest that kefir should contain a minimum of 10^7^ CFU/mL microorganisms and the final product should contain at least 10^4^ CFU/mL of yeast [3,10,12,13].

Kefir is a traditional fermented dairy beverage, originated from Caucasus Mountains, nowadays representing one of the most popular probiotic food consumed worldwide [2,3,4,7,13,14,15,16,17,18,19]. Currently, there is a renewed interest in this product [4,14]. Kefir is a stimulating and self-carbonated beverage produced through fermentation of different types of milk such as sheep, cow, goat, etc., [16,17]. Traditionally, kefir is produced by adding a starter culture termed “kefir grains” to milk. Kefir grains are composed of symbiotic lactose-fermenting yeasts, and non-lactose fermenting yeasts, as well as lactic acid bacteria (LAB), embedded within a polysaccharide and protein matrix called kefiran, which is reported to possess some antitumor activity [1,3,7,15,17,18,20,21]. Moreover, kefir consumption provides beneficial bacteria, yeast, vitamins, minerals, fatty acids, and complete protein which has been associated with numerous health-promoting benefits, including: anti-inflammatory, anti-carcinogenic, antimicrobial, and antidiabetic effects, lowering serum cholesterol levels, improved digestion and gut health, a reduction in hypertension, and regulation of reactive oxygen species [3,4,10,13,14,15,16,18,20,22]. Thus, kefir is a healthy nutrient-rich food that is beneficial for the anti-inflammation and immune system, and has been used as a supplement for patients with acquired immunodeficiency syndrome (AIDS), chronic fatigue syndrome (CFS), herpes, and cancer [3,4,22].

However, consumer demand for cow’s milk alternatives increased as a result of the increase in the diagnosis of lactose intolerance, allergies, and cholesterol issues. Furthermore, the ongoing trend of vegetarianism, with an increasing number of vegan/vegetarian consumers, has established a massive worldwide importance of non-dairy probiotic products [1,23]. Currently, alternative kefir formulations have been proposed, substituting milk and applying other carbohydrates to produce various non-dairy probiotic beverages of high added value which can be characterized as functional foods [7,13,16,17,21,24]. The new products might represent important foods providing live microorganisms to vegetarians with a limited availability of fermented products [1,13]. Likewise, interesting results have been presented regarding the application of kefir cultures for fermentation of molasses [9,13], rice [24], peanuts [16], cocoa pulp [7], soybean extracts [25], fruits and vegetables [1,2,12,13,14], hazelnut, walnut, rice, and soy plant milks [10,17,19,20,21].

Oil cakes/oil meals are the by-products obtained after oil extraction from the seeds. There are two types of oil cakes: edible and non-edible. Edible oil cakes are characterized by high nutritional value, i.e., their protein content ranges from 15% to 50% [26,27]. Their composition varies depending on their variety, growing conditions, and extraction methods. They are widely used for production of industrial enzymes, antibiotics, biopesticides, vitamins, and other biochemicals. Because of their rich protein content, they are used as animal feed supplement, especially for ruminants and fish [26]. Characterization of some oil cakes by the presence of molecules with high bioactivity might give an additional benefit to the experimental products, suggesting their production at large scale as healthy products, satisfying a wide range of consumers and showing a new way of plant products administration [1]. Flaxseed oil cake is a cheap by-product of flaxseed (*Linum usitatissimum* L.) oil pressing and is of a source of many bioactive substances such as proteins, fiber, and lignans. Many works have reported about positive influence of flaxseed consumption such as colon cancer prevention and reduction risk of cardiovascular disease. It is considered a “superfood” and generally recognized as safe (GRAS) [28]. Altogether, flaxseed is an excellent plant food candidate that meets the needs of the 21st century consumers in terms of being rich in nutrients as well as bioactive and functional ingredients [28,29,30]. However, some consideration in the application of various forms of flaxseed in human foods is related to the presence of antinutritional cyanogenic glycosides compounds (such as linustatin, neolinustatin, linamarin, lotaustralin) [28,31,32,33]. Thus, all of these antinutritive components must be removed or inactivated to physiologically undetectable limits to render flaxseed safe for consumption [28]. Various methods can be used to reduce or remove the cyanogenic glycosides compounds in the flaxseed, such as solvent extraction, biological (enzymatic), thermal or microwave treatment, and extrusion processing [28,31,32,33].

To the best of our knowledge there have been no reports about utilization of flaxseed oil cake obtained via cold press technique to produce fermented kefir like-beverages. Thus, the aim of the presented study is to produce beverage based on various concentrations of flaxseed oil cake fermented by kefir grains and evaluation of its bioactivity, microbiological, and physiochemical properties during refrigerated storage for 21 days.

## 2. Materials and Methods

### 2.1. Materials and Reagents

Flaxseed oil cake (FOC) obtained via cold press technique was kindly donated by ACS Sp. z o.o. (Bydgoszcz, Poland). According to manufacturer’s information the proximate composition of FOC was: solids content 80.50%, ash content 4.50%, protein content 41.97%, fat content 6.11%, carbohydrates 27.99%, fiber 6.29%. Commercial kefir grains (Yoghurt-Tek^®^, Lactoferm Kefir Series, Kefir-31) were obtained from Biochem s.r.l. (Rome, Italy). Sodium hydroxide, ammonia water, potassium iodide, silver nitrate, sulfuric acid, boric acid, hydrochloric acid, hydrogen peroxide, disodium phosphate, monosodium phosphate, phenolphthalein, 2,2-diphenyl-1-picrylhydrazyl (DPPH), 2,2′-azino-*bis*(3-ethylbenzothiazoline-6-sulfonic acid) (ABTS), methanol, Folin-Ciocalteu’s reagent, sodium carbonate, gallic acid, sodium nitrite, aluminum chloride, quercetin, 3,5-dinitrosalicylic acid, sodium tartrate tetrahydrate, acetic acid, sodium acetate, 2,6-dichlorophenolindophenol, oxalic acid, ascorbic acid, potassium ferricynide, trichloroacetic acid, ferric chloride, and chloramphenicol were purchased from Sigma Aldrich (Darmstadt, Germany). Glucose was supplied from Chempur (Piekary Śląskie, Poland). All reagents were of analytical grade. Buffered peptone water, MRS agar, and Sabouraud agar were obtained from Merck (Darmstadt, Germany).

### 2.2. Preparation of Flaxseed Oil Cake and Determiation of Cyanogenic Compounds

The preparation of FOC samples consisted of few steps. FOC was mixed with distilled water (*w*/*w*) to obtain three variants: A (5%), B (10%), C (15%). Then, the mixtures were heated at 90 °C for 1 h with constant stirring (250 rpm) and cooled down to room temperature. The samples were homogenized for 5 min with a homogenizer (SilentCrusherM, Heldolph, Germany) at 12,000 rpm. The influence of thermal processing on cyanogenic compounds content in FOC was performed according to Imran et al. [31] with a slight modification. Initially FOC samples were lyophilized (Beta 2-8 LSC Plus, Martin, Osterode am Harz, Germany). The samples (5 g) were taken in a Kjeldhal flask following the addition of 200 mL of distilled water and then slowly mixed. The sealed flask was incubated for 3 h at 30 °C to allow proper hydrolysis process of the sample mixture. After incubation, the distillation process was carried out by connecting the flask to a vapor distilling apparatus and the distillate was collected in a 250-mL flask containing 20 mL of 2.5% NaOH solution. Then, 8 mL of 6 M NH_4_OH and 2 mL of 5% KI solution were transferred into the distillate solution before titration against 0.02 M AgNO_3_ standard solution using a microburette. The amount of volume AgNO_3_ standard solution used for the titration was noted for each individual sample. Titration of a blank (distilled water) against AgNO_3_ standard solution was also run for comparison under the same room conditions. The determinations were carried out in triplicate. The following formula was used to calculate the total content (mg) of HCN (hydrogen cyanide), as given below:

$$\mathrm{HCN}=\mathrm{mL}\;\mathrm{of}\;0.02{\;M\;\mathrm{AgNO}}_{3}\;\times 1.08$$

Whereas HCN removal rate was calculated as:

$$\mathrm{HCN}\;\mathrm{removal}\;\mathrm{rate}\;\left(\%\right)=\left[1-\;\frac{\mathrm{HCN}\;\mathrm{content}\;\mathrm{of}\;\mathrm{FOC}\;\mathrm{after}\;\mathrm{heating}\;\left(\frac{\mathrm{mg}}{\mathrm{kg}}\right)}{\mathrm{HCN}\;\mathrm{content}\;\mathrm{of}\;\mathrm{FOC}\;\mathrm{without}\;\mathrm{heating}\;\left(\frac{\mathrm{mg}}{\mathrm{kg}}\right)}\;\right]\times 100$$

### 2.3. Fermentation

After homogenization the mixtures were dispensed into containers and were pasteurized by heating for 30 min at 60 °C, then cooled down and stored in a refrigerator day before the fermentation. Commercial kefir grains containing *Lactococcus lactis subsp. cremoris*, *Lactococcus lactis* subsp. *lactis* biovar *diacetylactis*, *Leuconostoc mesenteroides* subsp. *cremoris*, *Lactobacillus delbrueckii* subsp. *Bulgaricus*, and *Saccharomyces cerevisiae* were used in this study. Kefir-like beverages were produced by mixing 500 mL of a particular variant (pre-heated to 25 °C) with 5 g of kefir grains (containing 1.26 × 10^7^ ± 0.20 CFU/g of LAB and 1.36 × 10^7^ ± 0.34 CFU/g of yeast) and fermented at 25 °C in 100 mL closed sterile plastic cups for 24 h. After processing, beverages were cooled down and stored at 6 °C for 21 days. The analyses were performed after 1, 4, 7, 14, and 21 days of storage. The non-fermented reference samples were treated in the same way but without kefir grains addition, which served for comparison.

### 2.4. Determination of Total Solids Content (TSC), Protein Content (PC), Ash Content (AC), pH, and Titrable Acidity (TA)

Total solids (no. 968.11), protein (no. 99120), and ash (no. 94546) contents were measured according to AOAC standard methods [34]. A multiplication factor of 6.38 was used to convert nitrogen percentage to protein percentage. The pH of non-fermented and fermented samples were measured directly at 25 °C using a pH-meter (CP-411, Elmetron, Zabrze, Poland). The TA determination in the samples (expressed as g of lactic acid per 100 mL of the sample) was performed according to Bernat et al. [35], which consisted of mixing 5 g of sample with 20 mL of distilled water and titrated with 0.01 M NaOH solution, using phenolphthalein (0.1%, *w/v* in 95% ethanol) as an indicator.

### 2.5. Determination of Lactic Acid Bacteria and Yeast Viability

During the overall storage, samples (1 mL) were collected and diluted with 9 mL of sterile buffered peptone water (Merck, Darmstad, Germany), and serial dilutions were prepared. Lactic acid bacteria counts were determined on MRS (de Man, Rogosa and Sharpe) medium (Merck, Darmstad, Germany) after incubation at 37 °C under anaerobic conditions for 72 h, whereas yeast counts were determined on Sabouraud medium (supplemented with 150 ppm of chloramphenicol) at 25 °C for 72 h. The enumeration of microorganisms was performed in triplicate (by counting plates with 30–300 colonies) and the viable cell counts were expressed as CFU/mL of the samples.

### 2.6. Firmness and Viscosity Measurements

Texture profiles were performed at room temperature using a Zwick/Roell 2,5 Z equipment (Zwick/Roell, Ulm, Germany), equipped with a cylindrical probe (diameter 40 mm). The samples were carefully scooped into glass beakers (55 mm internal diameter and 70 mm high) in a depth of 50 mm. Penetration rate into the samples was 10 mm/s, and the penetration depth was 25 mm. From the results of the force-time, the firmness was calculated. The viscosity measurements were performed in a rheometer (AR G2, TA Instruments Ltd., New Castle, DE, USA). The samples were analyzed at 20 °C using a stainless steel cone plate having a diameter of 62 mm. Steady-state flow measurements were carried out at a shear rate 50 s^−1^ and the viscosity values were obtained from the TA Rheology Advantage Data Analysis equipment software V 5.4.7. (TA Instruments, New Castle, DE, USA).

### 2.7. Color Analysis

The samples were measured for color by a Konica Minolta CR - 5 colorimeter with the Hunter Lab color system (Konica Minolta, Osaka, Japan). Color coordinates were expressed as: lightness (L*), redness/greenness (+/− a*), and yellowness/blueness (+/− b*). Analyses were carried out at three independent times and presented as average values with ± standard deviation.

### 2.8. Preparation of Supernatants and Analysis of Antioxidant Activity

To obtain clear fluids for analyses the samples were transferred into 1.5-mL Eppendorf tubes and centrifuged at 14,000 rpm/min for 10 min at 20 °C (Centrifuge 5418 Eppendorf, Warsaw, Poland). The supernatants of the particular type of sample were mixed and filtered through 0.22-µm nylon membrane filters (Sigma-Aldrich, Darmstadt, Germany). The obtained clear fluids were used for further analyses. The DPPH radical scavenging activity was determined by method of Tong et al. [36]. Two milliliters of reaction mixture contained 1 mL of 0.1 mM DPPH methanolic solution and 1 mL of supernatants. The mixtures were shaken vigorously and placed in darkness. Absorbance was measured at 517 nm (UV-Vis Thermo Scientific Evolution 220 spectrophotometer, (Thermo Fisher Scientific Inc., Waltham, DE, USA) after 30 min and the inhibition ability was obtained from the formula:

$$\%DPPH\;inhibition=\left[1-\frac{Abs_{0}-Abs_{1}}{Abs_{0}}\right]\times 100$$

where Abs_0_ is absorbance without sample and Abs_1_ is absorbance in the presence of the sample.

The antioxidant activity of the samples was also measured by the ABTS radical cation decolorization assay following the methodology of Kim et al. [37]. The ABTS radical cation (ABTS^+^) was produced by reacting ABTS stock solution (7.4 mM in distilled water) with 2.45-mM potassium persulfate (final concentration), and allowing the mixture to stand in the dark at room temperature for 16 h before use. Prior to the assay, the solution was diluted in ethanol to give an absorbance of 0.700 ± 0.02 at 734 nm. A reagent blank reading was taken (A_0_). After adding 3 mL of diluted ABTS^·+^ solution to 50 µL of sample, the absorbance was measured at 734 nm after exactly 6 min of initial mixing (A_1_). The ABTS radical scavenging rate was calculated using the following formula:

$$\%ABTS\;inhibition=\left[1-\frac{Abs_{0}-Abs_{1}}{Abs_{0}}\right]\times 100$$

where Abs_0_ is the absorbance without sample and Abs_1_ is the absorbance in the presence of the sample.

### 2.9. Determination of Reducing Power

The reducing power of each sample was estimated by the method described by Tong et al. [36]. The supernatants (500 μL) were placed in a tube, to which 1.25 mL of phosphate buffer solution (0.2 M, pH 6.6), as well as 1.25 mL of 1% potassium ferricyanide solution were added. After incubation at 50 °C for 20 min, 1.25 mL of trichloroacetic acid solution were added to the tube. 1.25 mL of supernatant obtained by centrifugation at 3000 rpm for 10 min was diluted with 1.25 mL of deionized water. Finally, 0.25 mL of 0.1% ferric chloride solution was added to complete the assay. The absorbance was determined at 700 nm which represented the reducing power.

### 2.10. Determination of Total Polyphenolics Content (TPC)

The total polyphenolics content of each supernatant was determined by Folin-Ciocalteu method as described by Tong et al. [36]. The supernatants (100 µL) were mixed with 6 mL of distilled water and 0.5 mL of Folin-Ciocalteu’s reagent. After 3 min, 1.5 mL of saturated Na_2_CO_3_ solution was added and the mixture was incubated for 30 min in darkness at 40 °C. The absorbance of the mixture was measured at 765 nm (UV-Vis Thermo Scientific Evolution 220 spectrophotometer). The concentration of TPC was calculated as mg of gallic acid equivalents (GAE) per mL of sample (mg GAE/mL).

### 2.11. Determination of Total Flavonids Content (TFC)

The total flavonoids content (TFC) of each sample was estimated using the method described by Tong et al. with a slight modification [36]. 250 µL of supernatant was mixed with 1 mL of distilled water and 75 µL of 5% NaNO_2_ solution. After 5 min, 75 µL of 10% AlCl_3_ solution was added, and the mixture was allowed to stand for 6 min before the addition of 250 µL of 1 M NaOH. The total volume mixture was made up to 3 mL with distilled water, and then the absorbance was measured at 510 nm (UV-Vis Thermo Scientific Evolution 220 spectrophotometer). Quercetin was used for a calibration curve, and the results were expressed as mg of quercetin equivalents (QE) per mL of the sample (mg QE/mL).

### 2.12. Determination of Reducing Sugars Content (RSC)

The reducing sugars content (RSC) was determined by DNS (3,5-dinitrosalicylic acid) method. A total of 10 g of DNS was dissolved in 200 mL of distilled water by continuous stirring, then slowly 16 g of NaOH (dissolved first in 150 mL of distilled H_2_O) was added. The mixture was incubated at 50 °C with stirring to obtain a clear solution. Then 403 g of potassium sodium tartrate tetrahydrate was added in small portions. The mixture was filtered using a paper filter and the volume was made up to 1000 mL with distilled water. One milliliter of supernatant was mixed with 1 mL of 0.05 m acetate buffer (pH 4.8), and 3 mL of DNS reagent was added, then vigorously shaken. The mixtures were incubated in boiled water for 5 min then cooled at room temperature. Then, the absorbance values were recorded at 540 nm (UV-Vis Thermo Scientific Evolution 220 spectrophotometer). Glucose in acetate buffer was used for a calibration curve.

### 2.13. Determination of Ascorbic Acid Content

The Tillmans titration method involving a reduction of 2,6-dichlorophenolindophenol was used to determine the ascorbic acid content as previously described [38]. Two milliliters of supernatant was mixed with 2 mL of oxalic acid solution (2%) and vigorously shaken. The solution was quickly titrated with 2,6-dichlorophenolindophenol until pink color held for 30 s. The content of ascorbic acid was expressed as milligrams per mL of the sample.

### 2.14. Statistical Analysis

All data was expressed as mean ± standard deviation (SD). Statistical significance was determined using an analysis of variance (two-way ANOVA) followed by NIR Fisher test. The values were considered as significantly different when *p* < 0.05. All analyses were performed with Statistica version 10 (StatSoft Polska, Kraków, Poland).

## 3. Results and Discussion

### 3.1. The Removal of Cyanogenic Compounds as a Result of Thermal Treatment

The primary amount of cyanogenic compounds in flaxseed oil cake was 187.35 ± 8.34 mg/kg, which decreased to 11.15 ± 4.41 mg/kg (HCN removal rate 94.05% after incubating for 1 h at 90 °C). This reduction could be the result of the heat deactivation of glycosidase, the evaporation of HCN after formation from hydrolysis, or both. Heat induces the formation of HCN, so during thermal treatment, cyanogenic glycosides were hydrolyzed to HCN, and then evaporated. A similar reduction of cyanogenic compounds in flaxseed as a result of thermal treatment was previously reported, which is seen as a safe level for consumers [28,31,32,33]. Thus, it was concluded that after thermal processing, FOC is a safe raw material that can be used for fermentation.

### 3.2. The Changes of Total Solids Content, Protein Content, Ash Content, pH, and Titrable Acidity

As expected, the physicochemical properties of FOC were modified by the fermentation process. Average values of total solids content (TSC), protein content (PC), ash content (AC), pH, and titrable acidity (TA) are summarized in Table 1. After fermentation, the highest TSC was observed for sample C (10.63 ± 0.88%), whereas sample A was characterized by the lowest value (6.38 ± 0.82%). Similar values have been reported from hazelnut kefir [17]; whereas, on the contrary the TSC of all samples were lower than reported from pumpkin-based kefir beverage [12]. It was noted, that on day 4 of storage the TSC of all samples increased, whereas from day 7 a significant decrease was observed (*p* < 0.05). The protein content of the raw material as well as in the fermented samples was also investigated. It was found, that after fermentation, the PC values of samples A and C were lower than in raw material, higher PC was noticed in sample B (*p* < 0.05). However, those values were higher than reported for pumpkin puree kefir [12] and similar to hazelnut milk kefir [17]. The fluctuations in PC may result from slight protein synthesis by proliferation of microorganisms and synthesis of enzyme proteins or from a rearrangement of the composition following the degradation of other constituents [39]. It was found, that AC of fermented samples was significantly higher than the non-fermented ones (*p* < 0.05). This may be due to the kefir grains (5%) addition. The values were lower than reported from pumpkin-based kefir (4.69%) [12], but very similar to fermented hazelnut milk [17]. The highest AC was observed on day 21 for sample C (0.73 ± 0.01%), which is very close to the AC reported for semi-fat cow milk [17]. Although some fluctuations were observed during cold storage, on day 21, only value of sample A was lower than after fermentation. This observation is in contrary to the results of Obadina et al. who observed that the ash content of soymilk Nono increased gradually during 72-h fermentation, because of the reduction of certain chemical components such as carbohydrates, solids, and fat [40]. On the other hand, Hu et al. reported that AC of black soybean fermented with *Bacillus natto* decreased during fermentation [39]. However, on day 21 all samples were characterized by higher AC than the raw samples (*p* < 0.05). The fermentation significantly reduced pH to the range 3.84 ± 0.01 (A) – 4.18 ± 0.01 (C) in comparison to non-fermented samples (*p* < 0.05). The TA values of all samples significantly increased after fermentation, and an increasing tendency was observed during refrigerated storage. The highest TA (1.43±0.01 g of lactic acid per 100 mL of product) was observed on day 21 for sample C. The fluctuations of pH and TA values of all samples were observed over the storage period (*p* < 0.05). These changes were expected because of high viability of microorganisms over storage time. Decrease in pH and increase of TA values during fermentation using different substrates with kefir grains and other microorganisms (especially lactic acid bacteria) have been previously reported [2,10,12,17]. The acidity of fermented products is commonly maintained or decreased during storage, a fact that is attributed to microbial activity, which is a continuous fermentation process in which lactic acid bacteria or yeast assimilate available carbohydrates [10]. The reduction of pH is attributed to production of organic acids (mainly lactic acid), which during the fermentation of kefir is of great importance because of inhibitory effects against spoilage and pathogenic microorganisms [2]. In fact, a similar reduction of pH was reported from pumpkin puree kefir [12] as well as hazelnut milk [17], and, on the contrary, is much lower that the pH of tiger nut beverages (6.3–6.8), which is not sufficient for limiting the growth of food-borne pathogens [11].

### 3.3. The Lactic Acid Bacteria and Yeast Survivability during Cold Storage

With regard to microorganisms survivability, food matrix is considered as one of the pivotal factors regulating the colonization, since it might help to buffer the microorganisms through the stomach or might contain other functional ingredients (such as prebiotics), that could interact with them [41]. As listed in Table 2, the beverage formulations were an excellent matrix to develop non-dairy products, since LAB and yeast survivability was highly stable over storage period. At any time, the bacterial and yeast counts was maintained in the samples over the recommended for kefir level >10^7^ CFU/mL and >10^4^ CFU/mL (for bacteria and yeast, respectively) and are consistent with the previous reports of other authors using plant products to produce kefir-like beverages [10,14,17,42]. However, higher values were reported by Koh et al. (10^12^ CFU/mL and 10^9^ CFU/mL for LAB and yeast, respectively), who used pumpkin puree to develop kefir-like beverage, but in this study, the authors supplemented beverages with brown sugar [12]. According to Simova et al. LAB represents approximately 80–90%, whereas yeast represents 10–20% of the microbial counts in kefir products [42]. The microorganisms viability was the highest in sample B during cold storage; however on day 21 bacterial counts between samples were not statistically significant (*p* > 0.05), whereas significant differences in yeast counts were observed (*p* < 0.05). The fact that microorganisms in the samples remained highly concentrated might be due to the prebiotic effect of flaxseed fiber [10,35,41,43]. Indeed, HadiNezhad et al. demonstrated that flaxseed soluble dietary fiber acts as a good prebiotic, enhancing the LAB growth in kefir model [43]. Another important factor determining microorganisms viability and their metabolic activity is the product storage temperature [23,44,45]. The refrigerated storage generally increases the shelf life of fermented products and the survivability of microorganisms, maintaining their viability [44,46]. In fact, as the beverages were stored at low temperature (6 °C), the refrigerated conditions were presumably one of the main factors provided high LAB and yeast counts over a 21-day storage time.

### 3.4. The Change of Color

Table 3 presents the color parameters of both non-fermented and fermented samples over storage time. It was noticed, that fermentation significantly increased lightness (L*) values, decreased a* (redness) values, and increased b* (yellowness) value of A sample; whereas, values of samples B and C decreased (*p* < 0.05). It was noted that color parameters of fermented samples were affected during the storage time (*p* < 0.05). The color of fermented beverages is often related to the presence of pigments in the raw material, also, the variations in storage time and pH may affect the coloration of fermented foods [10]. The results are partially in agreement with the study of Randazzo et al. who noted that the fermentation of fruit juices with kefir cultures caused reduction of lightness and redness [14]. However, Santos et al. observed significantly increased lightness of fermented soymilk beverages [10].

### 3.5. Viscosity and Textural Changes

The apparent viscosity of the samples showed differences because of the FOC concentration as listed in Table 4. As the FOC concentration in the beverage increased, the apparent viscosity increased (*p* < 0.05). The results are higher than values reported for pumpkin puree kefir [12]. This may be explained by the content of flaxseed mucilage as well as protein content [17,43]. It was observed that after fermentation the viscosity of all samples significantly increased (*p* < 0.05). However, over the storage period various behavior of the samples was noticed. Since day 4 the viscosity of the sample A decreased significantly (*p* < 0.05) and remained constant until day 21. On the contrary, viscosity of sample B remained stable, whereas viscosity of sample C showed increasing tendency up to day 7 (5089.20 ± 2.49 MPa·s), which decreased until day 21 (*p* < 0.05). Table 4 shows also the firmness values of fermented and non-fermented samples. It was observed that the fermentation significantly enhanced the firmness of all samples (*p* < 0.05). The highest value was observed for sample C on day 14 (1.53 ± 0.24 N). The increase of firmness and viscosity of the samples may be linked with production of vicious polysaccharide kefiran [2,14].

### 3.6. The Changes of Reducing Sugars, Total Phenolic, Total Flavonoid, and Ascorbic Acid Contents

As presented in Table 5 there was a significant increase in RSC after fermentation in comparison to the non-fermented samples (*p* < 0.05). The increased RSC may be linked with enzymatic hydrolysis of polysaccharides such as flaxseed fiber by microorganisms, to obtain energy required for growth, thus generating higher amounts of polysaccharides derivatives [41]. However, since day 4 of cold storage, a decreasing tendency in RSC was observed in all samples (*p* < 0.05). This behavior is linked with the consumption of sugars by microorganisms, required for maintaining of metabolic activity and was reported in previous findings [2,7,9]. The total phenolic content (TPC), total flavonoid content (TSC), and ascorbic acid content (AAC) are summarized in Table 5. In general TPC and TFC increased after fermentation in comparison to non-fermented samples (*p* < 0.05). In this study, after fermentation, the highest TPC and TFC was detected in sample C (76.27 ± 0.11 mg GAE/mL and 13.68 ± 0.04 mg QE/mL, respectively). Observed TPC values were higher than reported from hazelnut kefir [17], but lower than in kefir produced from vegetable/fruit juices-based kefir beverages [1,14]. The TFC content was slightly lower than reported by Koh et al. for pumpkin kefir [12]. The increase of TPC as a result of fermentation have been previously reported [47]. However, fluctuations in TPC and TFC during cold storage were observed (*p* < 0.05). At the beginning of storage, the lowest TPC could have resulted from the metabolization of phenolic compounds by kefir microorganisms. The progress in the acidity of kefir samples especially at the end of storage, induced delinking of some phenolic compounds that were bonded to proteins, which might be linked with increased TPC [17,47]. After fermentation, a decrease of AAC was noticed (*p* < 0.05). Only on day 21 in sample C, a significantly higher AAC was observed (0.11 ± 0.01 mg/mL).

### 3.7. The Changes of Antioxidant Activity

The antioxidant activity has been reported to be concomitant with the development of reducing power [20,36]. As can be seen in Table 6, the RP of FOC increased significantly (*p* < 0.05) by fermentation. Same result has been reported by Liu et al. who observed a greater RP of milk-kefir and soymilk-kefir than that of the respective milks from which they were made [20]. In addition, the production of certain metabolites which demonstrate superior reducing power during kefir fermentation and react with free radicals to stabilize and terminate radical chain reactions has been suggested [20]. It is known that some lactic acid bacteria may exhibit excellent reducing power [16,48]. Moreover, some LAB strains are able to reduce the oxidative processes of dairy fermented products [49]. In fact, as can be seen in Table 6 the DPPH, ABTS scavenging activity and reducing power of raw samples significantly increased as a result of fermentation (*p* < 0.05). The highest DPPH radical inhibition was noticed for sample C (97.44 ± 0.26%), whereas the ABTS radical inhibition was found to be the highest in sample B (84.53 ± 0.44%). Significant differences in both radical scavenging systems were observed over storage period (*p* < 0.05). Those results are comparable with results of other authors who reported high antioxidant activity of fermented plant beverages [1,12,14,16,17,19]. It is interesting to note that fermented beverage from FOC did not lose its antioxidative properties as a consequence of the fermentation process, but, in fact, exhibited increased antioxidative activity. This observation is in line with studies showing that fermented foods derived from soya (such as tempeh, miso, and natto) as well as from peanut showed significantly higher levels of antioxidants than the non-fermented raw materials and were highly stable during storage time [20,47]. McCue and Shetty [47] and also Bensmira et al. [16] suggested that the increasing antioxidant activity during fermentation using kefir cultures may be due to the mobilization and production of individual phenolic compounds. However, according to Satir and Guzel-Seydim [22] as well as Liu et al. [20], kefir fermentation enhances the total antioxidant capacity, mainly because of the proteolysis activity of the microflora in the specified protein fractions. Thus, it is tempting to suggest that the antioxidant activity of developed beverages may be attributable in part to the production of phenolic compounds as well as the formation of bioactive peptides from FOC proteins.

## 4. Conclusions

Taking into account the increasing complexity of the needs of different typologies of consumers, including vegan/vegetarian and subjects with intolerance/allergy to dairy products, an approach in this work was applied to obtain kefir-like beverages from flaxseed oil cake substrate, using commercial kefir grains. As kefir grains possess healthy features, the study showed the way for their future employment in the fermentation industry, leading to products with high added value that are ready to be consumed. The microorganisms survivability and antioxidant activity over a 21-days storage period in refrigerated conditions made the beverages of high added value which can be characterized as functional foods. The beverages produced in this work may help to link the gap between the actual and an ideal and innovative consumption of plant products, recommended in human diet. Another key point to be noted is that the bioprocess utilizing oil cakes is attractive because of relatively cheaper availability of the oil cakes throughout the year, making it even more favorable when economic issues are considered.

## Figures and Tables

**Table 1:** Total solids content (TSC), protein content, ash content, pH, and titrable acidity (TA) of fermented beverages and unfermented (control) sample.

Sample *	Time of Storage (days)					
	Unfermented Sample	1	4	7	14	21
TSC (%)						
A	6.32 ± 1.95 ^Aa^	6.38 ± 0.82 ^Ba^	8.65 ± 2.81 ^Ca^	7.21 ± 1.35 ^Da^	4.82 ± 0.60 ^Ea^	1.01 ± 0.19 ^Fa^
B	10.33 ± 0.93 ^Ab^	9.94 ± 0.72 ^Bb^	11.44 ± 0.46 ^Cb^	8.09 ± 0.35 ^Da^	8.05 ± 0.80 ^Eb^	7.69 ± 0.00 ^Fb^
C	15.68 ± 3.53 ^Ac^	10.63 ± 0.88 ^Bb^	12.64 ± 1.86 ^Cc^	12.57 ± 0.68 ^Dc^	12.02 ± 0.66 ^Ec^	11.39 ± 0.53 ^Bc^
Protein content (mg/100 g)						
A	152.42 ± 1.57 ^Aa^	141.42 ± 0.29 ^Ba^	201.81 ± 0.77 ^Ca^	171.82 ± 0.15 ^Da^	217.26 ± 2.77 ^Ea^	290.13 ± 0.91 ^Fa^
B	284.90 ± 1.36 ^Ab^	299.90 ± 1.61 ^Bb^	332.27 ± 0.14 ^Cb^	231.21 ± 3.41 ^Db^	304.77 ± 3.65 ^Eb^	235.22 ± 0.64 ^Db^
C	526.13 ± 0.87 ^Ac^	431.88 ± 0.93 ^Bc^	479.26 ± 0.46 ^Cc^	551.55 ± 0.92 ^Dc^	516.69 ± 0.51 ^Ec^	453.52 ± 0.96 ^Fc^
Ash content (%)						
A	0.28 ± 0.01 ^Aa^	0.36 ± 0.01 ^Ba^	0.31 ± 0.01 ^Ca^	0.33 ± 0.01 ^Da^	0.35 ± 0.01 ^Ba^	0.31 ± 0.01 ^Ca^
B	0.48 ± 0.01 ^Ab^	0.54 ± 0.01 ^Bb^	0.50 ± 0.01 ^Cb^	0.50 ± 0.01 ^Cb^	0.53 ± 0.01 ^Bb^	0.55 ± 0.01 ^Bb^
C	0.52 ± 0.01 ^Ac^	0.69 ± 0.01 ^Bc^	0.63 ± 0.01 ^Cc^	0.67 ± 0.01 ^Dc^	0.69 ± 0.01 ^Bc^	0.73 ± 0.01 ^Ec^
pH						
A	6.71 ± 0.02 ^Aa^	3.84 ± 0.01 ^Ba^	4.05 ± 0.07 ^Ca^	3.78 ± 0.01 ^Da^	3.99 ± 0.01 ^Ea^	3.89 ± 0.01 ^Fa^
B	6.73 ± 0.01 ^Aa^	4.03 ± 0.01 ^Bb^	4.36 ± 0.01 ^Cb^	4.09 ± 0.01 ^Db^	4.58 ± 0.01 ^Eb^	4.16 ± 0.01 ^Fb^
C	6.63 ± 0.01 ^Ab^	4.18 ± 0.01 ^Bc^	4.33 ± 0.01 ^Cb^	4.14 ± 0.01 ^Db^	4.26 ± 0.01 ^Ec^	4.12 ± 0.01 ^Fc^
TA (g lactic acid/100 mL)						
A	0.05 ± 0.00 ^Aa^	0.54 ± 0.01 ^Ba^	0.74 ± 0.01 ^Ca^	1.17 ± 0.01 ^Da^	1.14 ± 0.03 ^Ea^	1.09 ± 0.02 ^Fa^
B	0.08 ± 0.00 ^Ab^	0.67 ± 0.01 ^Bb^	0.77 ± 0.02 ^Ca^	1.01 ± 0.01 ^Db^	0.90 ± 0.04 ^Eb^	1.22 ± 0.02 ^Fb^
C	0.09 ± 0.00 ^Ac^	0.68 ± 0.00 ^Bb^	1.17 ± 0.00 ^Cb^	1.25 ± 0.06 ^Da^	1.19 ± 0.01 ^Ec^	1.43 ± 0.01 ^Fc^

* Mixtures were produced with 5% (A), 10% (B), and 15% (C) of flaxseed oil cake. Values are means ± standard deviation of triplicate determinations. Means with different lowercase in the same column are significantly different at *p* < 0.05. Means with different uppercase in the same raw are significantly different at *p* < 0.05.

**Table 2:** Lactic acid bacteria (LAB) and yeast counts during storage time.

Sample *	Time of Storage (days)				
	1	4	7	14	21
LAB (CFU/mL)					
A	3.82 × 10^7^ ± 0.64 ^Aa^	6.43 × 10^7^ ± 0.04 ^Ba^	1.51 × 10^8^ ± 0.35 ^Ca^	2.38 × 10^7^ ± 0.40 ^Da^	2.64 × 10^7^ ± 0.43 ^Ea^
B	1.38 × 10^8^ ± 0.13 ^Ab^	2.05 × 10^8^ ± 0.26 ^Bb^	2.30 × 10^8^ ± 2.83 ^Cb^	1.06 × 10^8^ ± 0.14 ^Db^	3.25 × 10^7^ ± 1.91 ^Eb^
C	8.87 × 10^7^ ± 2.10 ^Ac^	4.68 × 10^7^ ± 0.32 ^Bc^	2.04 × 10^7^ ± 1.36 ^Cc^	6.36 × 10^7^ ± 0.40 ^Dc^	3.07 × 10^7^ ± 0.47 ^Ec^
Yeast (CFU/mL)					
A	2.69 × 10^6^ ± 0.84 ^Aa^	1.79 × 10^7^ ± 2.07 ^Ba^	3.25 × 10^6^ ± 0.50 ^Ca^	1.47 × 10^6^ ± 0.20 ^Da^	1.56 × 10^6^ ± 0.00 ^Ea^
B	6.64 × 10^8^ ± 4.75 ^Ab^	1.50 × 10^9^ ± 0.40 ^Bb^	1.43 × 10^8^ ± 0.11 ^Cb^	7.97 × 10^7^ ± 2.23 ^Db^	1.97 × 10^6^ ± 0.03 ^Eab^
C	6.09 × 10 ^6^± 2.57 ^Ac^	6.59 × 10^7^ ± 0.06 ^Bc^	3.86 × 10^6^ ± 0.50 ^Ca^	2.27 × 10^6^ ± 0.03 ^Dc^	2.41 × 10^6^ ± 0.19 ^Eb^

* Mixtures were produced with 5% (A), 10% (B), and 15% (C) of flaxseed oil cake. Values are means ± standard deviation of triplicate determinations. Means with different lowercase in the same column are significantly different at *p* < 0.05. Means with different uppercase in the same raw are significantly different at *p* < 0.05.

**Table 3:** Color values of fermented beverages and unfermented (control) sample.

Sample *	Time of Storage (days)					
	Unfermented Sample	1	4	7	14	21
*L* *						
A	34.51 ± 0.45 ^Aa^	63.37 ± 0.03 ^Ba^	51.21 ± 0.04 ^Ca^	63.59 ± 0.08 ^Da^	63.10 ± 0.08 ^Ea^	63.13 ± 0.05 ^Ea^
B	41.58 ± 0.03 ^Ab^	60.09 ± 0.04 ^Bb^	51.81 ± 0.02 ^Cb^	59.98 ± 0.05 ^Db^	59.44 ± 0.03 ^Eb^	59.93 ± 0.04 ^Db^
C	49.96 ± 0.02 ^Ac^	57.20 ± 0.02 ^Bc^	50.82 ± 0.01 ^Cc^	57.47 ± 0.05 ^Dc^	55.17 ± 0.06 ^Ec^	57.23 ± 0.06 ^Ec^
*a* *						
A	5.59 ± 0.01 ^Aa^	5.02 ± 0.01 ^Ba^	5.60 ± 0.02 ^Ca^	5.1 3± 0.01 ^Da^	5.17 ± 0.01 ^Ea^	5.17 ± 0.02 ^Fa^
B	6.63 ± 0.01 ^Ab^	5.50 ± 0.00 ^Bb^	5.55 ± 0.00 ^Cb^	5.44 ± 0.01 ^Db^	5.31 ± 0.01 ^Eb^	5.77 ± 0.01 ^Fb^
C	6.67 ± 0.01 ^Ac^	5.69 ± 0.01 ^Bc^	5.90 ± 0.01 ^Cc^	5.64 ± 0.01 ^Dc^	5.96 ± 0.01 ^Ec^	6.04 ± 0.01 ^Fc^
*b* *						
A	12.64 ± 0.07 ^Aa^	13.94 ± 0.02 ^Ba^	14.55 ± 0.01 ^Ca^	13.65 ± 0.02 ^Da^	13.50 ± 0.01 ^Ea^	13.04 ± 0.02 ^Fa^
B	15.39 ± 0.01 ^Ab^	14.16 ± 0.04 ^Bb^	13.74 ± 0.01 ^Cb^	14.18 ± 0.01 ^Db^	13.72 ± 0.08 ^Eb^	14.57 ± 0.04 ^Fb^
C	16.13 ± 0.02 ^Ac^	15.01 ± 0.01 ^Bc^	15.31 ± 0.01 ^Cc^	14.25 ± 0.01 ^Dc^	15.33 ± 0.08 ^Ec^	15.41 ± 0.04 ^Fc^

* Mixtures were produced with 5% (A), 10% (B), and 15% (C) of flaxseed oil cake. Values are means ± standard deviation of triplicate determinations. Means with different lowercase in the same column are significantly different at *p* < 0.05. Means with different uppercase in the same raw are significantly different at *p* < 0.05.

**Table 4:** Viscosity and firmness of fermented beverages and unfermented (control) sample.

Sample *	Time of Storage (days)					
	Unfermented Sample	1	4	7	14	21
Viscosity (MPa·s)						
A	244.05 ± 0.20 ^Aa^	545.44 ± 5.00 ^Ba^	112.47 ± 1.15 ^Ca^	101.74 ± 0.58 ^Da^	102.85 ± 2.05 ^Ea^	102.35 ± 0.06 ^Fa^
B	271.11 ± 0.25 ^Ab^	1051.20 ± 5.33 ^Bb^	1149.66 ± 3.46 ^Cb^	1115.40 ± 0.58 ^Db^	1001.25 ± 0.52 ^Eb^	1084.40 ± 4.50 ^Fb^
C	302.37 ± 0.52 ^Ac^	1075.78 ± 2.86 ^Bc^	2121.03 ± 2.31 ^Cc^	5089.96 ± 2.49 ^Dc^	4563.20 ± 0.46 ^Ec^	2969.15 ± 0.35 ^Fc^
Firmness (N)						
A	0.16 ± 0.02 ^Aa^	0.20 ± 0.02 ^Ba^	0.22 ± 0.36 ^Ca^	0.16 ± 0.14 ^Da^	0.40 ± 0.54 ^Ea^	0.45 ± 0.03 ^Fa^
B	0.25 ± 0.02 ^Ab^	0.27 ± 0.07 ^Bb^	0.34 ± 0.03 ^Ca^	0.39 ± 0.02 ^Da^	0.51 ± 0.05 ^Ea^	0.62 ± 0.95 ^Fab^
C	0.40 ± 0.02 ^Ac^	0.59 ± 0.01 ^Bc^	1.06 ± 0.13 ^Cc^	0.99 ± 0.02 ^Db^	1.53 ± 0.24 ^Eb^	1.13 ± 0.10 ^Fb^

* Mixtures were produced with 5% (A), 10% (B), and 15% (C) of flaxseed oil cake. Values are means ± standard deviation of triplicate determinations. Means with different lowercase in the same column are significantly different at *p* < 0.05. Means with different uppercase in the same raw are significantly different at *p* < 0.05.

**Table 5:** Reducing sugars (RSC), total polyphenolics (TPC), total flavonoids (TFC), and ascorbic acid (AAC) contents of fermented beverages and unfermented (control) sample.

Sample *		Time of Storage (days)				
	Unfermented Sample	1	4	7	14	21
	RSC (mg/mL)					
A	6.16 ± 0.01 ^Aa^	10.50 ± 0.01 ^Ba^	7.75 ± 0.01 ^Ca^	7.29 ± 0.01 ^Da^	3.92 ± 0.00 ^Ea^	4.12 ± 0.00 ^Fa^
B	6.15 ± 0.02 ^Aa^	10.51 ± 0.00 ^Ba^	9.83 ± 0.01 ^Cb^	7.87 ± 0.00 ^Db^	5.67 ± 0.00 ^Eb^	5.22 ± 0.00 ^Fb^
C	7.90 ± 0.00 ^Ab^	16.24 ± 0.02 ^Bb^	14.32 ± 0.01 ^Cc^	13.84 ± 0.01 ^Dc^	11.00 ± 0.01 ^Ec^	10.34 ± 0.01 ^Fc^
	TPC (mg GAE/mL)					
A	26.88 ± 0.03 ^Aa^	32.90 ± 0.08 ^Ba^	30.17 ± 0.04 ^Ca^	37.66 ± 0.08 ^Da^	39.59 ± 0.00 ^Ea^	48.87 ± 0.03 ^Fa^
B	41.91 ± 0.15 ^Ab^	42.67 ± 0.06 ^Bb^	36.74 ± 0.06 ^Cb^	41.89 ± 0.03 ^Db^	35.24 ± 0.04 ^Eb^	38.73 ± 0.00 ^Fb^
C	57.16 ± 0.15 ^Ac^	76.27 ± 0.11 ^Bc^	71.37 ± 0.21 ^Cc^	65.67 ± 0.08 ^Dc^	75.68 ± 0.18 ^Ec^	73.93 ± 0.15 ^Fc^
	TFC (mg QE/mL)					
A	6.03 ± 0.00 ^Aa^	6.26 ± 0.03 ^Ba^	5.48 ± 0.00 ^Ca^	6.13 ± 0.00 ^Da^	6.40 ± 0.00 ^Ea^	6.11 ± 0.03 ^Fa^
B	10.16 ± 0.03 ^Ab^	10.95 ± 0.03 ^Bb^	10.84 ± 0.00 ^Cb^	9.55 ± 0.00 ^Db^	10.83 ± 0.15 ^Eb^	11.20 ± 0.03 ^Fb^
C	11.74 ± 0.03 ^Ac^	13.68 ± 0.04 ^Bc^	14.49 ± 0.00 ^Cc^	15.88 ± 0.03 ^Dc^	13.28 ± 0.03 ^Ec^	16.27 ± 0.03 ^Fc^
	AAC (mg/mL)					
A	0.05 ± 0.01 ^Aa^	0.05 ± 0.00 ^Ba^	0.04 ± 0.01 ^Ba^	0.04 ± 0.01 ^Ba^	0.05 ± 0.01 ^Aa^	0.06 ± 0.01 ^Aa^
B	0.06 ± 0.01 ^Aba^	0.04 ± 0.01 ^Bb^	0.05 ± 0.02 ^BCb^	0.06 ± 0.01 ^Cb^	0.06 ± 0.01 ^Cab^	0.06 ± 0.01 ^Ca^
C	0.07 ± 0.00 ^Ab^	0.05 ± 0.01 ^Bb^	0.07 ± 0.00 ^Ab^	0.07 ± 0.01 ^ACb^	0.07 ± 0.00 ^Ab^	0.11 ± 0.01 ^Dc^

* Mixtures were produced with 5% (A), 10% (B), and 15% (C) of flaxseed oil cake. Values are means ± standard deviation of triplicate determinations. Means with different lowercase in the same column are significantly different at *p* < 0.05. Means with different uppercase in the same raw are significantly different at *p* < 0.05.

**Table 6:** Reducing power (RP), DDPH, and ABTS radical scavenging ability of fermented beverages and unfermented (control) sample.

Sample *		Time of Storage (days)				
	Unfermented Sample	1	4	7	14	21
	RP (%)					
A	65.60 ± 0.08 ^Aa^	77.15 ± 0.06 ^Ba^	84.03 ± 0.10 ^Ca^	86.88 ± 0.05 ^Da^	89.80 ± 0.10 ^Ea^	98.45 ± 0.08 ^Fa^
B	81.60 ± 0.08 ^Ab^	76.65 ± 0.13 ^Bb^	78.75 ± 0.06 ^Cb^	81.10 ± 0.06 ^Db^	86.70 ± 0.08 ^Eb^	92.45 ± 0.00 ^Fb^
C	92.73 ± 0.05 ^Ac^	95.88 ± 0.10 ^Bc^	91.28 ± 0.10 ^Cc^	96.28 ± 0.06 ^Dc^	94.05 ± 0.05 ^Ec^	91.75 ± 0.10 ^Fc^
	DPPH inhibition (%)					
A	50.52 ± 0.68 ^Aa^	76.21 ± 0.01 ^Ba^	79.02 ± 0.06 ^Ca^	78.33 ± 0.31 ^Da^	79.47 ± 0.02 ^Ea^	83.68 ± 0.06 ^Fa^
B	80.52 ± 0.23 ^Ab^	94.88 ± 0.00 ^Bb^	95.25 ± 0.00 ^Cb^	96.32 ± 0.06 ^Db^	97.56 ± 0.00 ^Eb^	95.13 ± 0.00 ^Fb^
C	85.98 ± 0.00 ^Ac^	97.44 ± 0.26 ^Bc^	96.71 ± 0.00 ^Cc^	94.64 ± 0.00 ^Dc^	97.02 ± 0.07 ^Ec^	97.47 ± 0.47 ^Fc^
	ABTS inhibition (%)					
A	68.13 ± 0.58 ^Aa^	77.43 ± 0.05 ^Ba^	83.95 ± 0.06 ^Ca^	81.98 ± 0.13 ^Da^	78.05 ± 0.06 ^Ea^	77.20 ± 0.26 ^Fa^
B	68.60 ± 0.39 ^Ab^	84.53 ± 0.44 ^Bb^	83.35 ± 0.26 ^Cb^	80.90 ± 0.45 ^Db^	84.20 ± 0.22 ^Eb^	78.85 ± 0.44 ^Fb^
C	69.78 ± 0.39 ^Ac^	76.58 ± 0.26 ^Bc^	79.35 ± 0.13 ^Cc^	87.60 ± 0.76 ^Dc^	79.45 ± 0.24 ^Cc^	78.63 ± 0.53 ^Dc^

* Mixtures were produced with 5% (A), 10% (B), and 15% (C) of flaxseed oil cake. Values are means ± standard deviation of triplicate determinations. Means with different lowercase in the same column are significantly different at *p* < 0.05. Means with different uppercase in the same raw are significantly different at *p* < 0.05.
